# ‘BLUES’ procedure for assessing the blue level of the sclera in Osteogenesis Imperfecta

**DOI:** 10.1186/s13023-024-03192-z

**Published:** 2024-04-27

**Authors:** Valerio Di Martino, Fabiana Mallone, Alessandro Lambiase, Mauro Celli, Alice Mannocci, Luca Celli, Pietro Mangiantini, Pasquale Fino, Antonietta Moramarco

**Affiliations:** 1 Department of Sense Organs, Sapienza University of Rome, Viale del Policlinico 155, Rome, 00161 Italy; 2 Department of Pediatrics and Child Neuropsychiatry, Sapienza University of Rome, Rome, Italy; 3 Department of Public Health and Infectious Diseases, Sapienza University of Rome, Rome, Italy; 4 Department of Clinical Internal, Anesthesiologic and Cardiovascular Sciences, Sapienza University of Rome, Rome, Italy

**Keywords:** Osteogenesis Imperfecta (OI), Blue sclera, ‘BLUES’ procedure, Computed analysis, Diagnosis, Rare diseases

## Abstract

**Purpose:**

Blue sclera is a characteristic and common clinical sign of Osteogenesis Imperfecta (OI). However, there is currently no widely accepted, objective method for assessing and grading blue sclera in individuals with OI.

To address this medical need, this study is aimed to design and validate a new method called ‘BLUES’ (BLUe Eye Sclera) to objectively identify and quantify the blue color in the sclera of patients affected by OI.

**Methods:**

Sixty-two patients affected by OI and 35 healthy controls were enrolled in the present prospective study, for a total of 194 eyes analyzed.

In the 'BLUES' procedure, eye images from patients with OI and control subjects were analyzed to assess and grade the blue level of the sclera using Adobe Photoshop Software. The validation process then involved comparing the results obtained with the ‘BLUES’ procedure to the judgement of experienced ophthalmologists (JEO).

A receiver-operating characteristic (ROC) curve analysis was used to examine the overall discriminatory power. The sensitivity and specificity levels and the Cohen's Kappa (K) indexes of ‘BLUES’ and ‘JEO’ were estimated versus the standard OI diagnosis. The K indexes of ‘BLUES’ versus ‘JEO’ were also evaluated.

**Results:**

The optimal cut-off point of the scleral blue peak was calculated at 17%. Our findings demonstrated a sensitivity of 89% (CI95%: 0.835–0.945) and specificity of 87% (CI95%: 0.791–0.949) for the ‘BLUES’ procedure with an agreement versus the diagnosis of OI of 0.747. In comparison, the sensitivity and specificity of ‘JEO’ ranged from 89 to 94% and 77% to 100%, respectively, with an agreement ranging from 0.663 to 0.871 with the diagnosis of OI. The agreement between ‘BLUES ‘and ‘JEO’ evaluations ranged from 0.613 to 0.734.

**Conclusions:**

Our findings demonstrated an 89% sensitivity and an impressive 87% specificity of our method to analyze the blue sclera in OI. The results indicated high agreement with disease diagnosis and were consistent with evaluations by experienced ophthalmologists. The ‘BLUES’ procedure appears to be a simple, reliable and objective method for effectively identify and quantify the blue color of the sclera in OI.

## Background

Osteogenesis Imperfecta (OI) is a rare genetic disease that affects the connective tissue and is the most common herile bone fragility disorder in children [1–3]. This condition is characterized by reduced bone mass and skeletal fragility, resulting in significant morbidity, including chronic pain, limited mobility, skeletal deformities, and growth deficiency. The decreased bone mass associated with OI increases susceptibility to fractures, which can occur with minimal trauma or in unusual locations [1–3]. The estimated incidence of OI is approximately 1 in 10,000 individuals. In approximately 85–90% of cases, the disease is associated with dominantly inherited mutations in either the COL1A1 or COL1A2 genes, which encode type I collagen [3, 4]. OI manifests with a broad spectrum of clinical manifestations that range from subtle increase in fracture frequency to progressively deforming and perinatal lethal forms [4].

An underexplored domain in OI pertains to eye-related complications, wherein type I collagen assumes a pivotal role as a structural component within the eye. Ocular conditions, such as blue sclera, refractive errors, corneal thinning, keratoconus, glaucoma, cataracts, and retinal detachment, may manifest as consequences of underlying connective tissue abnormalities [5–7]. Specifically, blue discoloration of the sclera is the main clinical finding among ocular manifestations in OI. This results from both the loss and structural alteration of collagen fibrils, leading to an increase in the translucency of the sclera and the exposure of the underlying choroid [8].

In OI, the blue color of the sclera has yet to undergo objective evaluation and quantification. To date, the sole method employed for identifying and grading blue sclera in OI relies on subjective assessment by physicians and visual comparison with a standard commercial blue color scale. This evaluation is conducted through direct observation of the eye and the use of slit lamp examination [9]. However, this procedure lacks objectivity, and depends on the individual observer's experience. Interestingly, a computed analysis to assess blue sclera color was performed in a pilot study for the diagnosis of iron deficiency [10], showing good sensitivity and specificity. However, despite these encouraging findings, the application of computerized scleral color grading remains unexplored in OI.

Therefore, the purpose of this study is to design and validate a simple, reliable and objective method to identify and quantify the blue color of the sclera in OI. This method was called ‘Blue Eye Sclera (BLUES) procedure’.

## Methods

The ‘BLUES’ procedure is a color grading method that objectively evaluates the blue sclera by analyzing an eye image through the use of Adobe Photoshop software. In this prospective, case-controlled, and single-center study, 62 patients with a diagnosis of OI and 35 healthy control subjects matched for age, gender and race, were consecutively enrolled between July 2022 to May 2023 at the Department of Sense Organs, Sapienza University of Rome, Italy. The study was prospectively reviewed by the Ethics Committee of the Sapienza University of Rome (Prot. N. 0096/2023). The research followed the Tenets of the Declaration of Helsinki and the Good Clinical Practice Recommendations. A written informed consent was obtained from all subjects and from parents in case of minor age.

This study consists of three phases: sample selection, development and validation of the procedure.

### Sample selection

Two groups were recruited for the study: one group of patients affected by OI and the other consisting of healthy controls. The OI group included 62 patients (32 males and 30 females) aged 14 to 70 years (mean age: 30 ± 17 years). Among these OI patients, 50 (80.6%) had the COL1A1 mutation, while 12 (19.4%) had the COL1A2 mutation, confirmed by molecular genetic testing.

All patients were classified as Type I OI according to the Sillence classification [11]. Exclusion criteria were: history of Marfan or Ehler–Danlos syndrome, blood iron deficiency or long-term minocycline use or any other conditions causing blue sclera, ocular surgery, trauma or infection.

Enrolled healthy controls were 35, including 18 males (51%) and 17 females (49%), between 17 and 52 years of age (mean age: 28 ± 9 years). These healthy subjects were recruited from outpatients of the eye clinic according to the exclusion criteria.

All participants in the study were Caucasian in ethnicity.

The mean refractive errors were evaluated in both OI patients and controls and expressed as spherical equivalent.

### Development of the procedure

The 'BLUES' procedure was developed through two sequential steps: (i) capturing a photograph of the sclera; (ii) analyzing the photograph using Adobe Photoshop software. These steps were always performed by the same physician, who was not involved in the subsequent validation of the procedure.

**Fig. 1:**
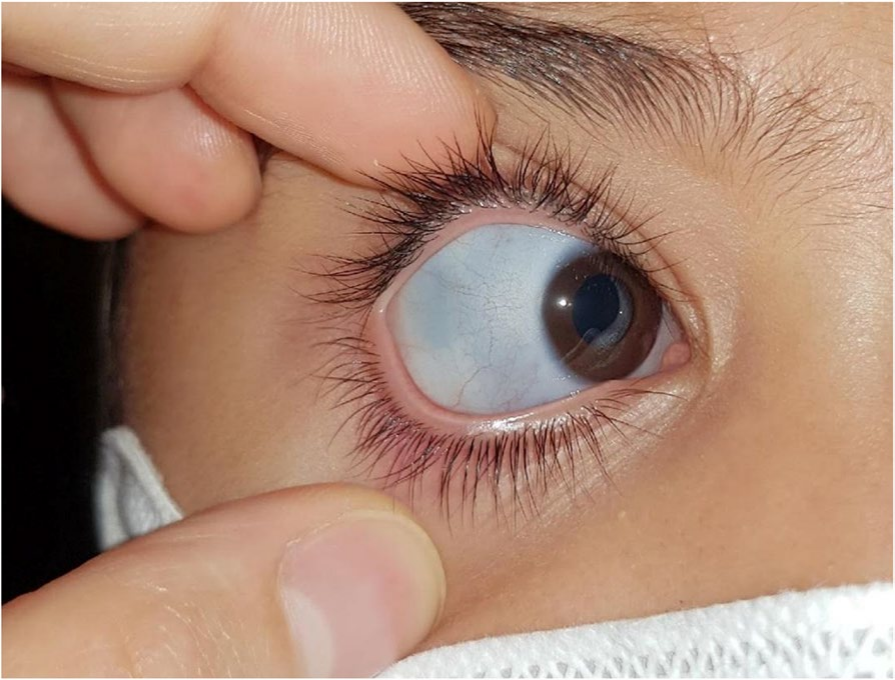
Photo acquisition of the sclera

The first step involved capturing photographs (Fig. 1) under consistent environmental conditions to ensure reproducibility and objectivity for subsequent comparisons. Subjects were positioned in the same room, with identical backgrounds and lighting conditions. Specifically, all photographs were captured under dark lighting conditions with the use of a light-emitting diode (LED) flashlight.

The same Samsung Galaxy S8 smartphone camera (12 MP, f/1.7, 26 mm (wide), 1/2.55", 1.4 µm, dual pixel PDAF, OIS) was used for each examination to maintain consistency. We opted for a smartphone device over a professional camera to enable widespread use of this diagnostic tool.

**Fig. 2:**
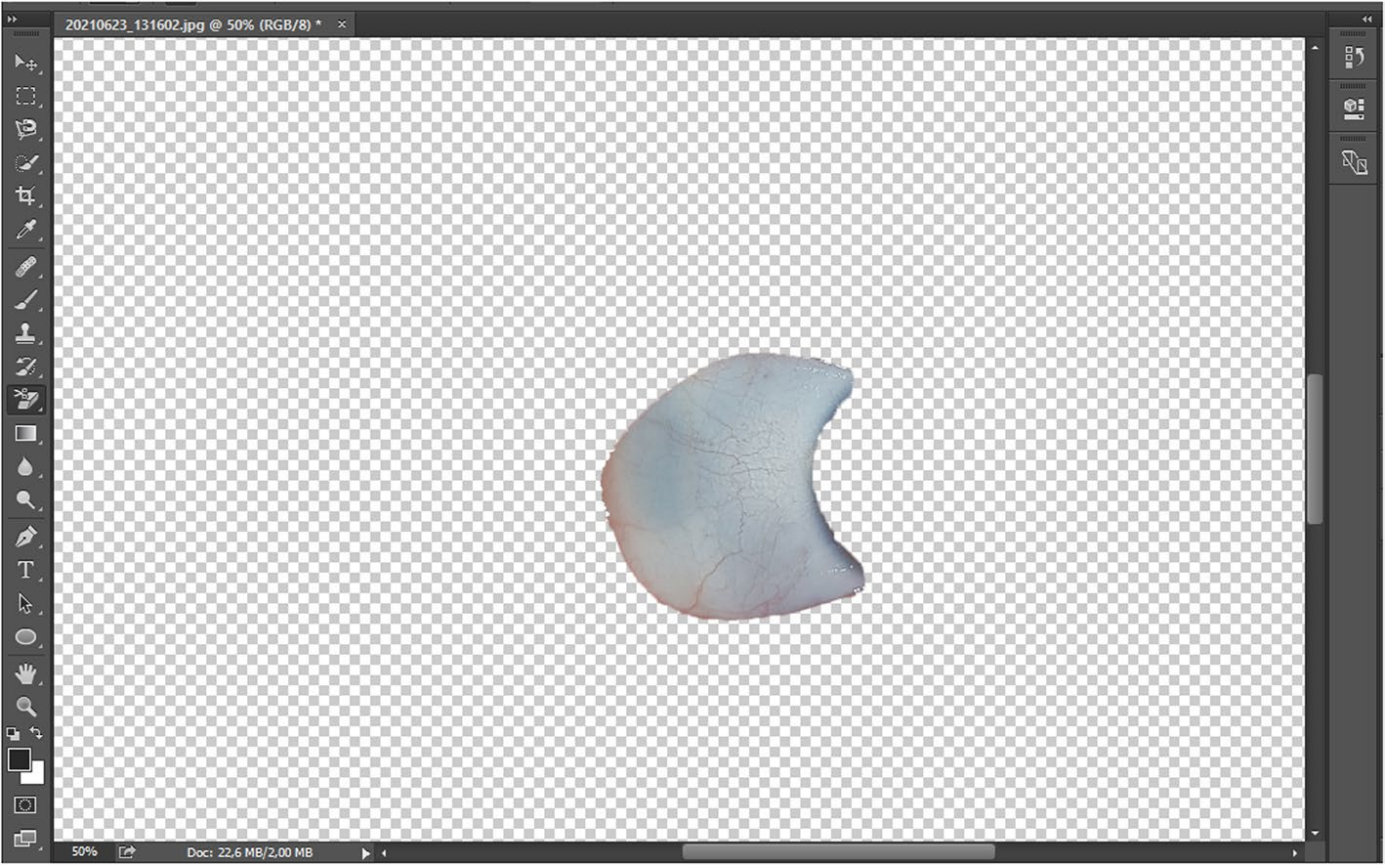
Cut level of the temporal sclera in Adobe Photoshop software

**Fig. 3:**
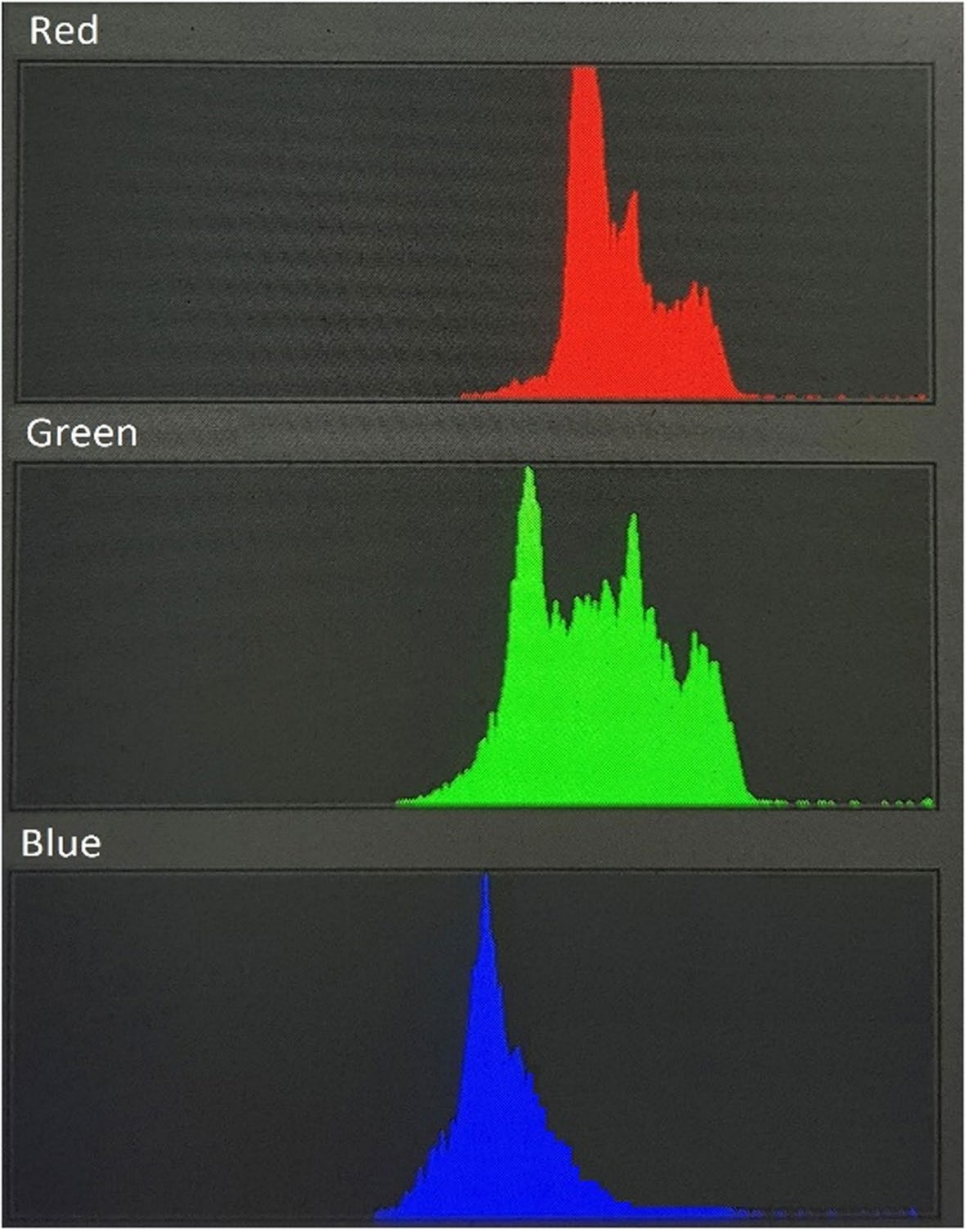
RGB color distribution curves in Adobe Photoshop software

**Fig. 4:**
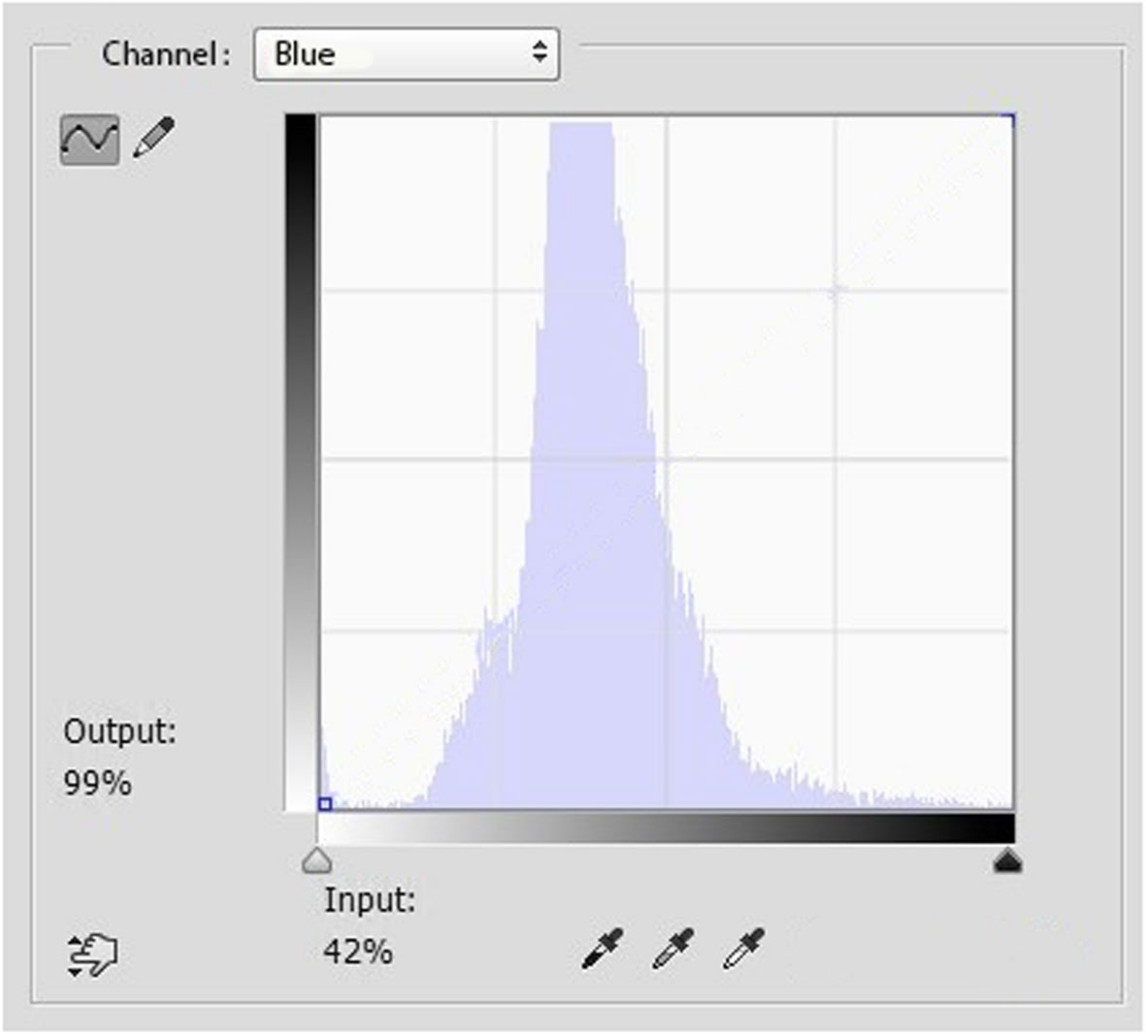
Adobe photoshop Image showing the peak distribution of the blue color (in this selected case: 42%) with reference to the total scleral area

**Fig. 5:**
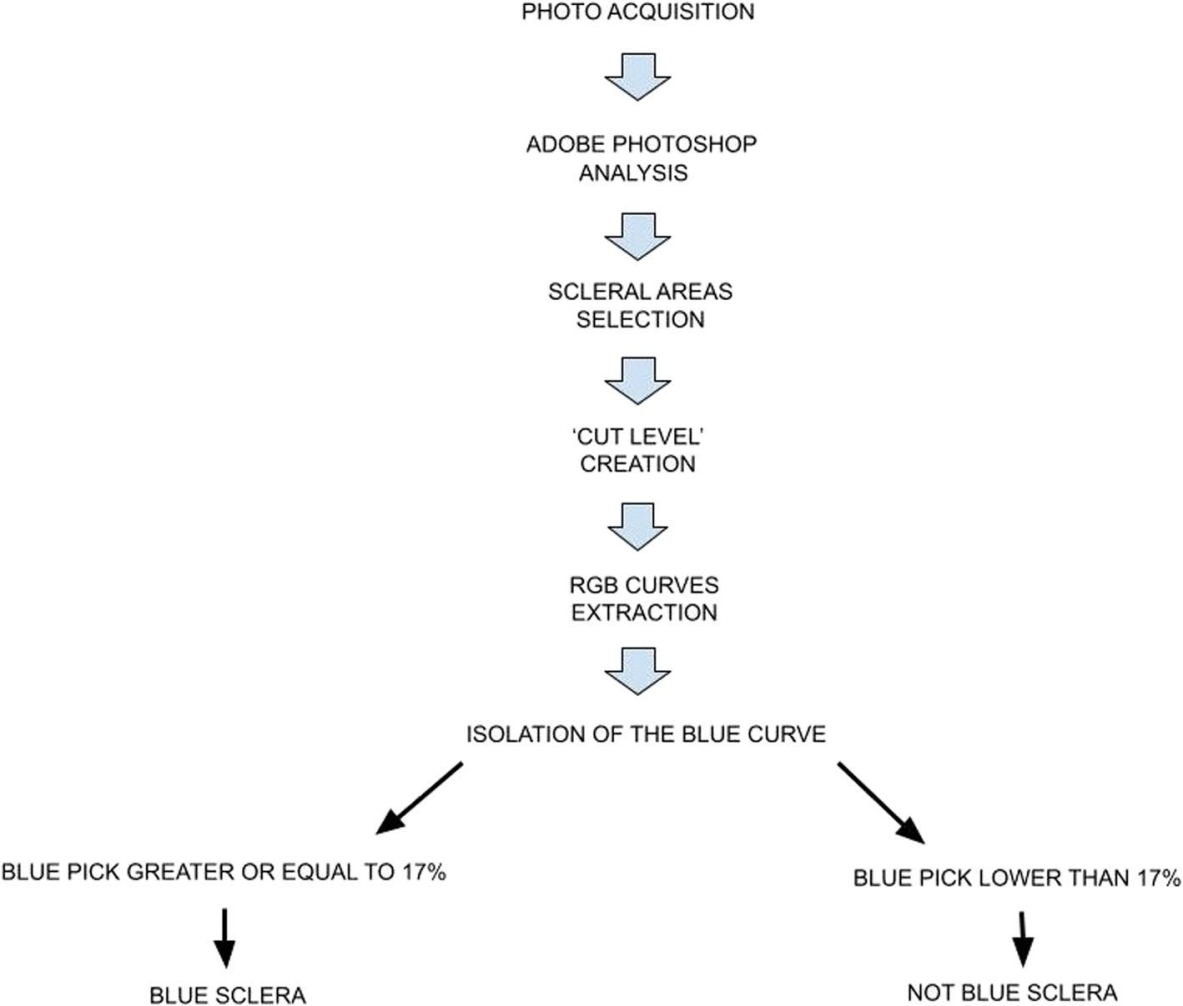
Flowchart showing the key steps of the BLUES procedure

All photographs were then analyzed using Adobe Photoshop software. The areas corresponding to the sclera were manually selected and extracted, creating a secondary level referred to as the 'cut level' (Fig. 2). Subsequently, RGB (red–green–blue) color distribution curves were extracted from the 'cut level' (Fig. 3). From these curves, we isolated the blue curve and used it to determine the maximum concentration of the blue color, expressed as a whole number percentage (% of the blue peak) (Fig. 4). The key steps of the BLUES procedure are summarized in Fig. 5.

### Validation of procedure

After the development of the ‘BLUES’ procedure, we proceeded to validate the method to verify its reliability. This validation process involved comparing the results obtained from Adobe Photoshop software with the assessment of the blue sclera made by qualified ophthalmologists. This approach was termed ‘Judgment of Expert Ophthalmologists (JEO)’. In the ‘JEO’ evaluation, four ophthalmologists (called observers) with an expertise in rare eyes diseases were commissioned to evaluate the photos of the sclera: (i) all observers had over 15 years of experience in the field of rare diseases in Ophthalmology, (ii) each observer was tasked to determine whether the sclera in the photos appeared blue or white, (iii) the photo evaluations were conducted individually, utilizing identical Liquid Crystal Display (LCD) monitors, within the same room, and under consistent dim light conditions.

### Statistical analysis

A sample size exceeding 50 patients (97 individuals, totaling 194 eyes) was enrolled, as usually recommended to ensure robust statistical power and to demonstrate a concordance coefficient of at least 0.70 [12]. Collected variables included the ‘JEO’ evaluations as a binary variable indicating the color of the sclera (blue/white), and the percentage of blue determined by Adobe Photoshop software. The receiver operating characteristic (ROC) curve was constructed using values of blue percentage obtained through the’BLUES’ procedure in OI patients and healthy groups to determine the cut-off value. To determine the accuracy of the ‘BLUES’ procedure, the area under the ROC curve (AUC) was calculated.

The Kappa Cohen’s index (K) was computed in order to measure the of inter-rater reliability between the ‘BLUES’ and ‘JEO’ assessments versus the diagnosis of OI. The agreement between the’BLUES’ procedure and each ‘JEO’ observer was also calculated. The benchmarks for Kappa coefficient according to Altman et al. were [13]: poor agreement from 0.00 to 0.20; fair agreement from 0.21 to 0.40; moderate agreement from 0.41 to 0.60; good agreement from 0.61 to 0.80; very good agreement greater than 0.80.

The data was collected in Excel file. SPSS 26 for Windows software was used to perform the statistical analysis. A statistically significant difference in testing results was indicated by *p* < 0.05.

## Results

A total of 194 photos of the sclera were collected.

**Fig. 6:**
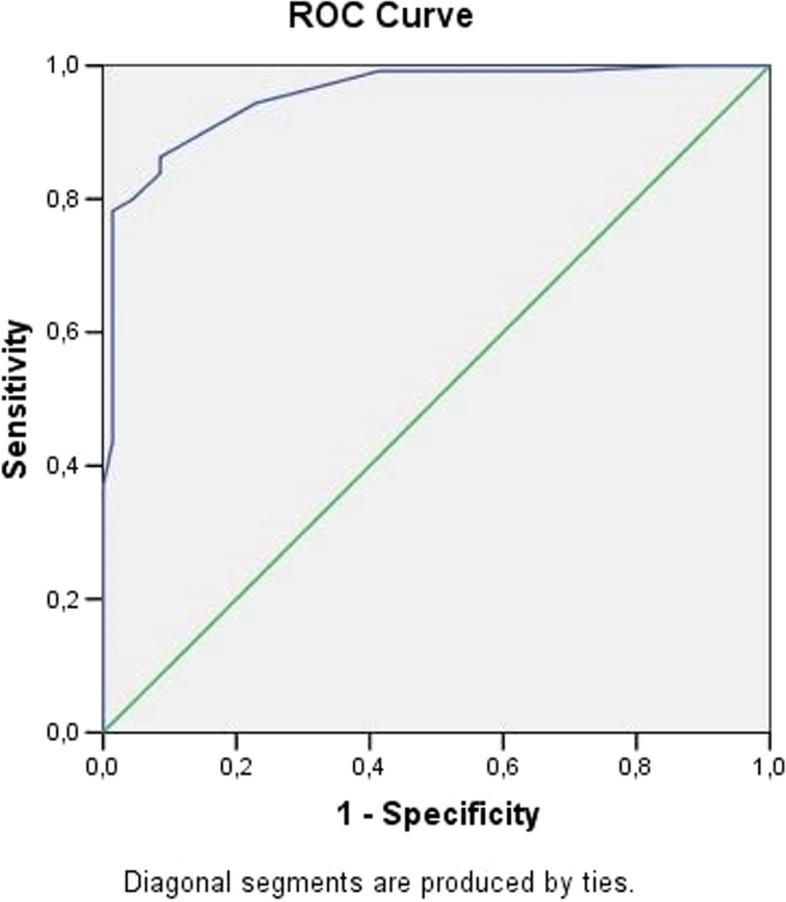
ROC curve (AUC = 0.96)

The AUC reached 0.96. The corresponding ROC curve results are graphically presented in Fig. 6.

The optimal cut-off point of the scleral blue peak was calculated at 17%. The sclera was defined as blue if the percentage of the blue peak was greater than or equal to 17%, while it was defined ‘not blue sclera’ if lower than 17%.

The sensitivity of the ‘BLUES’ procedure resulted in 89% (CI95%: 0.835–0.945) and the specificity 87% (CI95%: 0.791 -0.949). The ‘BLUES’ procedure detected the blue sclera in 110 out of 124 of patients affected by OI (89%: true positive) while non-blue sclera was detected in 61 out of 70 healthy controls (87%: true negative).

In Table 1, the sensitivity, specificity, and Kappa index analyses between the 'BLUES', ‘JEO’ procedures and the diagnosis of OI are reported.

**Table 1:** Sensibility, specificity and agreement analysis for the BLUES and JEO diagnostic approaches

Methodologies			*Diagnosis*		
			*OI* *N = 124* n(col%) ^a^	*Controls* *N = 70* n(col%)	*Kappa index*
***BLUES***		Blue (%) Not blue (%)	110 (89 ^b^) 14 (11)	9 (13) 61 (87^c^)	0.747
***JEO***	***Observer 1***	Blue (%) Not blue (%)	112 (90 ^b^) 12 (10)	0 (0) 70 (100 ^c^)	0.871
	***Observer 2***	Blue (%) Not blue (%)	117 (94 ^b^) 7 (6)	10 (14) 60 (86 ^c^)	0.808
	***Observer 3***	Blue (%) Not blue (%)	110 (89 ^b^) 14 (11)	16 (23) 54 (77 ^c^)	0.663
	***Observer 4***	Blue (%) Not blue (%)	116 (94 ^b^) 8 (6)	12 (17) 58 (83 ^c^)	0.774

^a^column percentage

^b^sensibility

^c^specificity

The Kappa index of ‘BLUES’ procedure was reported as 0.747. The observer n.1 resulted to have sensitivity = 90% and specificity = 100% and with a Kappa index of 0.871. The observer 2 reported a sensitivity = 94% and a specificity = 86% and with a Kappa index of 0.808. The observer 3 resulted in sensitivity = 89% and specificity = 77% with a Kappa index of 0.663. The observer 4 reported a sensitivity = 94% and a specificity of 83%, with a Kappa index of 0.774.

The agreement between the ‘BLUES’ procedure and each of the four ‘JEO’ observers is reported in Table 2. The agreement between the ‘BLUES’ and observer n.1 was 85% on both blue sclera and non-blue sclera, with a Kappa index of 0.690. Agreement between ‘BLUES’ and observer n.2 was 93% for blue sclera and 79% for non-blue sclera, with Kappa index of 0.734. Agreement between ‘BLUES’ and observer n.3 was 88% for blue sclera and 72% for non-blue sclera, with Kappa index of 0.613. Agreement between ‘BLUES’ and observer n.4 was 90% for blue sclera and 72% for non-blue sclera, with Kappa index of 0.633. Notably, the mean spherical equivalent values were not different between OI group and controls (-1.04 ± 1.72 Vs -1.41 ± 3.22; *p* = 0.110).

**Table 2:** Agreement between observers and BLUES procedure

		***BLUES***	***Kappa index***
*Observer 1*	agreement blue agreement not blue	85% 85%	0.690
*Observer 2*	agreement blue agreement not blue	93% 79%	0.734
*Observer 3*	agreement blue agreement not blue	88% 72%	0.613
*Observer 4*	agreement blue agreement not blue	90% 72%	0.633

## Discussion

The results obtained from this study reveal an 89% sensitivity and 87% specificity of our method to analyze blue sclera in OI. The ‘BLUES’ procedure appears to be a simple, reliable and objective method for effectively identifying and quantifying the blue color of the sclera, a distinctive feature of patients with OI.

The evaluation of blue sclera color is a crucial clinical indicator in diagnosing OI, particularly in pediatric cases. Additionally, scleral color emerges as a significant criterion for distinguishing different subtypes of OI, with potential prognostic implications. This is particularly the case when distinguishing types I and IV of the Sillence classification [11].

To date, the sole method is to identify the scleral color of the eye with slit lamp examination and compare it with different scales. In their original work, Sillence et al. (1993) employed successive dilutions of a custom-made ink to establish a spectrum of colors. While this approach guaranteed internal consistency within the study, reproducing the findings proved challenging for others who lacked access to the same dye range [14]. Later, a standardized scale was established to describe scleral eye colors in individuals with OI using the Munsell color system [9]. The Munsell color system is internationally acknowledged in the paint and printing industries for characterizing surface colors [9]. The established scale for blue sclera color in OI includes the following measurements: hue at 2.5 PB, chroma at 2, and a value range from 0 to 10 [9].

However, this comparative method is prone to variation, lacks objectivity, and relies on the subjective experience of the individual observer. Recently, a computed analysis evaluating the blue coloration of the sclera was conducted in a pilot study aimed at diagnosing iron deficiency [10]. The results showed promising sensitivity and specificity at 78.4% and 50%, respectively. Despite these positive findings, the utilization of computerized scleral color grading has yet to be investigated in the context of OI.

To address this medical need, we performed a multi-step study to develop and validate a computer-based procedure, the ‘BLUES procedure’, to identify and quantify the blue color of the sclera in OI.

In our study, the ’BLUES procedure’ demonstrated 89% sensitivity and an impressive 87% specificity. These results were comparable to those obtained with the JEO evaluation which showed 90%, 94%, 89%, and 94% sensitivity for ophthalmologists 1, 2, 3, and 4, respectively, along with specificities of 100%, 86%, 77%, and 83% for the same ophthalmologists. Additionally, our findings demonstrated superior outcomes in comparison to those reported by Hervé Lobbes et al. with sensitivities of 60.8%, 45.1%, and 29.4% for physicians 1, 2, and 3, respectively, and specificities of 68.8%, 81.3%, and 93.8% for physicians 1, 2, and 3, respectively. It's worth noting that the Authors in that study relied on the judgment of three non-ophthalmologist physicians [10].

The 'BLUES' procedure demonstrated a diagnostic agreement of 0.747 with OI, consistent with the 'JEO' approach, which exhibited a concordant agreement range of 0.663 to 0.871.

Furthermore, our assessment extended to evaluating the agreement between the 'BLUES' procedure and each 'JEO' observer regarding blue sclera in OI patients, showing a Kappa index ranging from 0.613 to 0.734. These findings highlight a high level of reliability and consistency between the two procedures in the assessment of blue sclera in OI.

Therefore, the ‘BLUES’ procedure emerges as a simple, reliable and objective method to identify and quantify the blue color of the sclera in OI. However, the ‘BLUES’ procedure certainly has some limitations. First of all, the exclusive Caucasian ethnicity of all patients poses a limitation to the generalizability of the procedure.

Moreover, we don’t know if Adobe Photoshop analysis would produce the same results regardless of the type of smartphone used. Also, patient compliance is a major limitation in ‘BLUES’ procedure as it is not always possible to acquire satisfactory photos.

## Conclusions

In this project, we aimed to design and validate a simple, reliable and objective method for detecting and measuring the blue sclera color in OI. The ‘BLUES’ procedure allows to overcome the subjectivity of direct examination of the sclera in OI, and its assessment could serve as an inexpensive and effective diagnostic tool in general ophthalmology and primary care. This method, incorporating a computed grading system for scleral blue color, is expected to support an early diagnosis and classification of OI, including the differentiation between OI subtypes. Furthermore, the widespread availability of this tool would enable additional assessments including investigating scleral color variations over time and exploring a potential relationship between the intensity of blue hue and disease severity. However, further efforts are mandatory to substantiate the validity of the ‘BLUES’ procedure in a large population of individuals with OI. Additionally, there is potential to expand this assessment to encompass cases of scleral blue discoloration unrelated to OI.

## Data Availability

The data used to support the findings of this study are not openly available due to reasons of sensitivity and are available from the corresponding author upon reasonable request. Data are located in controlled access data storage at Sapienza University.

## Abbreviations

OI: Osteogenesis Imperfecta
BLUES: BLUe Eye Sclera
JEO: Judgment of Expert Ophthalmologists
K: Cohen's Kappa
